# Phylogenetic studies of *Prunella strophiata* (Passeridae: *Prunella*) based on complete mitochondrial DNA sequences

**DOI:** 10.1080/23802359.2016.1181994

**Published:** 2016-07-22

**Authors:** Xiaonan Sun, Zhonglou Sun, Dingzhen Liu, Wenliang Zhou

**Affiliations:** a School of Life Sciences, Anhui University, Hefei, Anhui, China;; b Ministry of Education Key Laboratory for Biodiversity Science and Ecological Engineering, College of Life Sciences, Beijing Normal University, Beijing, China

**Keywords:** Mitochondrial genome, Passeriformes, phylogenetic relationships, *Prunella strophiata*

## Abstract

Rufous-breasted Accentor (*Prunella strophiata*) is a small-sized bunting with an extremely geographical range in the world. Here, the complete mitochondrial genome of *P. strophiata* (16,830 bp in length) has been analyzed for building the database. Similar to the typical mtDNA of vertebrates, it contained 37 genes (13 protein-coding genes, two rRNA genes and 22 tRNA genes) and a non-coding region (D-loop). Overall base composition of the complete mitochondrial DNA is A (30.3%), G (14.7%), C (31.0%) and T (24.0%), the percentage of A and T (54.3%) is higher than G and C (45.7%). All the genes in *P. strophiata* were distributed on the H-strand, except for the ND6 subunit gene and 9 tRNA genes which were encoded on the L-strand. The phylogenetic relationships of 12 Passeriformes species were reconstructed based on the complete mtDNA sequences using the Bayesian inference method.

Rufous-breasted Accentor (*Prunella strophiata*) is a small-sized passerine bird, which is reported to be locally frequent in Pakistan and China, common in northern India, fairly common in Nepal and common in Bhutan (Grimmett et al. 1998; Zheng 2002). The population is suspected to be stable in the absence of evidence for any declines or substantial threats. Currently, it has been categorized as Least Concern (LC) by IUCN. Up to now, only partial sequence of the mitochondrial DNA of Rufous-breasted Accentor is available. Considering the small size, maternal inheritance, accelerated rate of mutation compared to the nuclear DNA and little or no recombination which was useful for phylogenetic relationships at several taxonomic levels (Brown et al. 1979; Ballard & Whitlock 2004), we analyze the complete mitochondrial genome of *P. strophiata* for preserving the commercial and biodiversity value and reconstructing the phylogeny.

An adult Rufous-breasted Accentor was collected from Shaanxi Foping National Nature Reserve, which is numbered 20151201 and stored at the museum in the School of Life Sciences, Anhui University, China. The genomic DNA was extracted from the muscle tissue using standard phenol-chloroform methods (Sambrook & Russell 2001). The complete mtDNA sequences of *P. strophiata* (accession no. KU975800) is 16,830 bp in length which was sequenced by High-throughput sequencing technology. It encode 37 genes totally containing 13 protein-coding genes (PCGs) and 22 tRNA genes, two rRNA genes and non-coding region (D-loop). All the genes in *P. strophiata* were distributed on the H-strand, except for the ND6 subunit gene and nine tRNA genes which were encoded on the L-strand. Overall base composition of the complete mitochondrial DNA is A (30.3%), G (14.7%), C (31.0%) and T (24.0%), which indicated a strong AT bias (Shadel & Clayton 1997).

In order to convince the mitochondrial sequence obtained in this study, we selected 12 species from the Passeriformes order to reconstruct the phylogenetic tree with Bayesian inference (BI) method using the MrBayes version 3.1 (Huelsenbeck & Ronquist 2001). The best-fitting nucleotide substitution model (GTR + I + G) was selected by MrModeltest version 2.1 (Nylander 2004). The Markov Chain Monte Carlo (MCMC) was run with four chains for 1,000,000 generations until the average standard deviation (SD) of split frequencies reached a value less than 0.01. Bayesian posterior probabilities calculated from the sample points after the MCMC algorithm had started to converge (Zhan & Fu 2011). The phylogenetic tree is classified into five clades (Figure 1). The first lineage, containing four species (*Montifringilla nivalis*, *M. adamsi*, *M. taczanowski* and *Pyrgilauda blanfordi*), belongs to genus *Montifringilla*. The second lineage, genus *Motacilla*, includes three species (*Motacilla cinerea*, *M. alba* and *M. lugens*). The third group, only contains *Euphagus cyanocephalus*. The forth group, genus *Lonchura*, contains *Lonchura striata* and *L. punctulata.* The last group, genus *Prunella*, sister group to *Lonchura*. We expect that the present result can contribute to construct molecular identification of this species and be helpful to explore the phylogeny of Passeriformes.

**Figure 1.**
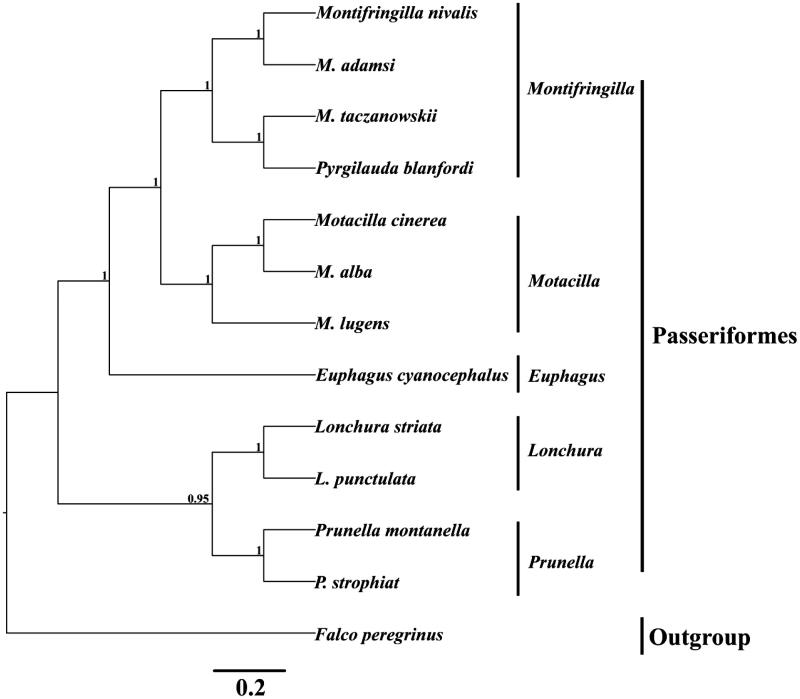
Inferred phylogenetic relationships of 12 Anatidae species were reconstructed based on the complete mtDNA sequences using Bayesian inference (BI). Numbers at each node indicate percentages of Bayesian posterior probabilities (BPPs). GenBank accession numbers for the published sequences are NC_025911 (*Montifringilla nivalis*), NC_025913 (*Montifringilla adamsi*), NC_025914 (*Montifringilla taczanowskii*), NC_025912 (*Pyrgilauda blanfordi*), NC_027933 (*Motacilla cinerea*), NC_029229 (*Motacilla alba*), KU246035 (*Motacilla lugens*), NC_018827 (*Euphagus cyanocephalus*), NC_029475 (*Lonchura striata*), NC_028036 (*Lonchura punctulata*), NC_027284 (*Prunella montanella*), KU975800 (*Prunella strophiat*) and NC_000878 (*Falco peregrinus*).
